# Draft genome sequence of fish pathogenic *Enterococcus faecalis* GIFTE22 isolated from diseased Nile tilapia (*Oreochromis niloticus*)

**DOI:** 10.1128/mra.00416-26

**Published:** 2026-05-18

**Authors:** Santu Biswas, Md. Rony Babu, Farzana Yeasmin, Promi Saha, Arpita Guha, Md. Mustafizur Rahman, Tofazzal Islam, Md. Mahbubur Rahman

**Affiliations:** 1 Institute of Biotechnology and Genetic Engineering, Gazipur Agricultural University 198780, Gazipur, Bangladesh; 2 Infectious Diseases Division, International Centre for Diarrhoeal Disease Research Bangladesh 56291, Dhaka, Bangladesh; Montana State University, Bozeman, Montana, USA

**Keywords:** *Enterococcus faecalis*, Nile tilapia, genome sequence, streptococcosis, virulence factors

## Abstract

Here, we describe the draft genome of fish pathogenic *Enterococcus faecalis* GIFTE22 isolated from skin lesion of diseased Nile tilapia in Bangladesh. The genome comprises 2,773,187 bp with a guanine-cytosine (GC) content of 37.5%. It contains 2,699 genes, three antibiotic resistance genes, and 19 virulence-associated genes.

## ANNOUNCEMENT

*Enterococcus faecalis*, a gram-positive pathogen associated with diseases in plants, humans, and animals (1–3), causes streptococcosis in tilapia and other fishes in Bangladesh, Egypt, and India (3–8). This report describes the draft genome sequence of a fish pathogenic *E. faecalis* GIFTE22.

GIFTE22 was isolated from the skin lesion of diseased Nile tilapia collected from a farm at Jolarpar (24.051358, 90.369493), Gazipur, Bangladesh. Infected fish showed protruded and opaque eyes, excessive mucus discharge, skin discoloration and erosions, abdominal distension, and irregular swimming. A swab from the lesion was inoculated and streaked on *Streptococcus agalactiae*-selective agar base (HiMedia, India) with 6% sheep blood and incubated at 35°C for 24–48 h. A single colony was picked and repeatedly streaked on same agar to obtain the pure isolate. GIFTE22 appeared as cream-white, circular colonies, and was Gram-positive, catalase- and oxidase-negative, glucose-fermentative, non-motile, bile esculin hydrolysis-positive, tolerant to 6.5% NaCl, and positive for indole, methyl red and Voges-Proskauer tests (2). Intraperitoneal injection of 100 μL bacterial suspension (1.2 × 10^7^ CFU/mL) into healthy tilapia reproduced same clinical symptoms as naturally infected fish, with 90% mortality (our unpublished data).

Genomic DNA was extracted from overnight culture (Todd Hewitt Broth, 37°C) using DNeasy Blood & Tissue Kit (Qiagen). After DNA quality and quantity assessment (8), libraries were prepared via Illumina DNA Prep Kit and sequenced on an Illumina NextSeq 2000 platform generating 300 cycles (2 × 150 bp paired-end reads).

A total of 1,437,542 paired-end raw reads were quality-checked using FastQC v0.12.1 (9), followed by trimming with Trimmomatic v0.39 (10). Draft genome assembly was performed using high-quality reads with SPAdes v4.2.0 (11). Genome annotation was performed using Prokaryotic Genome Annotation Pipeline (PGAP) v6.10 (12) from NCBI. Genome completeness was evaluated by CheckM v1.2.4 (13). Taxonomic classification was supported by database comparison and confirmed by NCBI Average Nucleotide Identity (ANI) analysis (14). Antibiotic resistance genes (ARGs) were annotated by CARD v4.0.1 (15) and ResFinder v4.7.2 (16), virulence factors via VFDB (17), plasmids with PlasmidFinder v2.0.1 (18), and functional subsystems with RAST v2.0 (19). Default parameters were used for all software unless otherwise specified.

The genome size of GIFTE22 was 2,773,187 bp with 37.5% GC content. Genome completeness covers 99.6% with 0.38% contamination. ANI showed 98.7% identity with the type strain *Enterococcus faecalis* ATCC 19433. *De novo* assembly resulted in 55 contigs. The N50, L50, and largest contig size were 301.1 kbp, 4, and 541,917 bp, respectively. PGAP recognized a total of 2,699 genes, 2,617 protein coding sequences (CDs), 62 rRNAs, 54 tRNAs, and four ncRNAs. The final genome coverage was approximately 149.4×. RAST dentified a total of 239 annotated subsystems. Three high-confidence ARGs (*efrB*, *efrA*, *dfrE*, *lsaA*, *emeA*, *VanT*) and 19 virulence-associated genes were predicted, including adherence (*ace*, *epbA-C*, *ecbA*, *efcA*), antiphagocytosis (*cps* cluster), biofilm formation (*bopD*, *fsr*, *gelE*, *sprE*), toxin (*cylR2*). No plasmid sequence was detected.

## Data Availability

The Whole-Genome Shotgun projects of the *E. faecalis* strain GIFTE22 have been deposited in GenBank under accession number JBSXVY000000000. The versions described in this paper are the first versions. The raw sequencing reads have been deposited in the NCBI Sequence Read Archive (SRA) under accession number SRR36540873 associated with BioSample accession number SAMN53802719.
